# Velocity and Directionality of the Electrohysterographic Signal Propagation

**DOI:** 10.1371/journal.pone.0086775

**Published:** 2014-01-21

**Authors:** Lasse Lange, Anders Vaeggemose, Preben Kidmose, Eva Mikkelsen, Niels Uldbjerg, Peter Johansen

**Affiliations:** 1 Department of Engineering, Faculty of Science and Technology, Aarhus University, Aarhus, Denmark; 2 Department of Obstetrics and Gynecology, Aarhus University Hospital, Aarhus, Denmark; John Hunter Hospital, Australia

## Abstract

**Objective:**

The initiation of treatment for women with threatening preterm labor requires effective distinction between true and false labor. The electrohysterogram (EHG) has shown great promise in estimating and classifying uterine activity. However, key issues remain unresolved and no clinically usable method has yet been presented using EHG. Recent studies have focused on the propagation velocity of the EHG signals as a potential discriminator between true and false labor. These studies have estimated the propagation velocity of individual spikes of the EHG signals. We therefore focus on estimating the propagation velocity of the entire EHG burst recorded during a contraction in two dimensions.

**Study Design:**

EHG measurements were performed on six women in active labor at term, and a total of 35 contractions were used for the estimation of propagation velocity. The measurements were performed using a 16-channel two-dimensional electrode grid. The estimates were calculated with a maximum-likelihood approach.

**Results:**

The estimated average propagation velocity was 2.18 (±0.68) cm/s. No single preferred direction of propagation was found.

**Conclusion:**

The propagation velocities estimated in this study are similar to those reported in other studies but with a smaller intra- and inter-patient variation. Thus a potential tool has been established for further studies on true and false labor contractions.

## Introduction

Premature birth, defined as birth before 37 completed weeks of gestation, is a serious obstetric challenge which is associated with a high occurrence of neonatal morbidity and mortality. In industrialized countries, preterm delivery is responsible for 70% of mortality and 75% of morbidity in the neonatal period. [1] Also, it contributes to significant long term neurodevelopmental problems, pulmonary dysfunction and visual impairment. [1], [2]

Although important insights into the pathophysiology of preterm labor have been achieved over the past several decades, effective therapeutic interventions to decrease spontaneous preterm delivery have not been established. [3] On the contrary, the preterm birth rate in the United States has increased from 9.4% in 1981 to 12.8% in 2006, although the latest reports show a slight decrease to 12.2% in 2009. [4] A recent study estimated the worldwide preterm birth rate at 9.6%. [5]

The current therapeutic treatment for threatening preterm labor has two goals: To delay the actual delivery and to optimize fetal status before preterm delivery. In the effort to delay delivery, several methods are used. These include administration of anti-contraction medications (tocolytic agents), antibiotics when infection is suspected, and strengthening of the uterine cervix (cervical cerclage) in women with a weakened cervix. [6], [7] The delay of delivery, combined with the use of corticosteroids, helps prevent neonatal respiratory distress syndrome by accelerating the fetal lung maturity.

Today it is difficult to identify which women will benefit from the above mentioned treatments. This is mainly due to the fact that it is difficult to make a distinction between true labor contractions and Braxton Hicks contractions, which are sporadic uterine contractions that occur in false labor. The inability to make a distinction between the two types of contractions, often leads to pregnant women with Braxton Hicks contractions being over-treated, and women in preterm labor being undertreated.

The methods most commonly used to assess contractions include tocodynamometry and intrauterine pressure (IUP) catheters. Unfortunately, these methods have some major drawbacks. The tocodynamometer suffers from an inherent lack of accuracy, seriously limiting its prognostic value. [8] An IUP catheter offers a much better accuracy, but is an invasive method and can therefore only be used on women in active labor. At the same time they increase the risk of infection or accidental induction of labor since it involves rupture of the fetal membranes [8] and has in rare cases been associated with uterus perforation and placental abruption. [9] As such, no appropriate technique of assessing uterine contractions exists today, highlighting the need for a new and improved non-invasive method.

Over the years, an alternative method of estimating uterine activity has emerged, based on monitoring of the electrical activity in the smooth muscle cells of the uterus known as the myometrium. This noninvasive measurement of electrical activity in the uterus is commonly referred to as an electrohysterogram (EHG). The signals measured by an EHG represent the aggregated electrical activity in the underlying smooth muscle cells. They reflect the continuous de- and repolarization of the cell membranes, and therefore also the contraction of the muscle cells, making it a good marker for uterine activity. [10]–[12] The uterine electrical activity appears in bursts, each corresponding to a contraction. A burst is seen as a low frequency (<1 Hz) oscillating signal that can last more than a minute. [13] The individual peaks in the signal are commonly referred to as “spikes” in the literature.

Studies suggest that the characteristic properties of the EHG changes as delivery approaches. [14]–[16] Among these, the peak frequency of the power density spectrum (PDS) has been one of the most predictive EHG parameters in both human and animal studies. [15], [17] It has been shown that laboring preterm patients have a significantly higher PDS peak frequency than non-laboring patients. [18] Other parameters related to the timing and amplitude of the EHG bursts have demonstrated less potential in the prediction of true labor. [19]

The newest area of research in relation to EHG measurements are concerned with the propagation of the electrical signal in the myometrium. Studies have shown that the number of gap junctions in the uterus increase close to delivery. [20], [21] The increasing number of gap junctions form an electrical syncytium required for coordination of myometrial cells for effective contractions. We would expect that this in turn leads to an increased propagation velocity (PV) of electrical signals in the myometrium before delivery. As a result it has been suggested that EMG recordings can be used to asses PV in vivo and thereby determine the stage of pregnancy. [17]

Some studies have been performed in an effort to estimate the propagation velocity with the use of surface EHG. In one case, Lucovnik et al. estimated the velocity with the use of a bipolar electrode setup, taking into account only one dimension of propagation at a time. [17] In other studies, Rabotti et al. used a 64-channel high density two-dimensional electrode grid. [22], [23] This permits estimation of both velocity components, for a small area of the uterus. All of these studies are based on the estimation of time delays between spikes. In this paper, we focus therefore on estimating the propagation velocity of the entire EHG bursts that occur during a contraction. This means that the velocity estimates are calculated from entire bursts corresponding to a full contraction event. Since the origin of a contraction is a priori unknown, a secondary objective of this study is to investigate the direction of propagation of the EHG signals, and thereby also the origin of the contractions.

## Materials and Methods

The propagation velocity and the direction of propagation are estimated on EHG signals from laboring women. This section describes the procedure for obtaining the measurements, the measuring equipment that was used as well as the signal processing that was applied to the acquired signals.

The measurements were carried out at the Department of Obstetrics and Gynecology at Aarhus University Hospital, Denmark. The measurements procedure was approved by The Central Denmark Region Committees on Health Research Ethics (permission number: 32939). The study complies with the Helsinki II declaration. Measurements were performed on six women in labor at term after signing an informed written consent. All women gave birth within 11 hours from the time of measurement. The elapsed time between the measurement and time of delivery can be seen in Table 1 for all patients.

**Table 1:** Elapsed time between time of measurement and delivery.

*Patient #*	*1*	*2*	*3*	*4*	*5*	*6*
Elapsed time	5 h56 min	3 h44 min	5 h46 min	7 h12 min	10 h12 min	8 h1 min

**Figure 1.Electrode:**
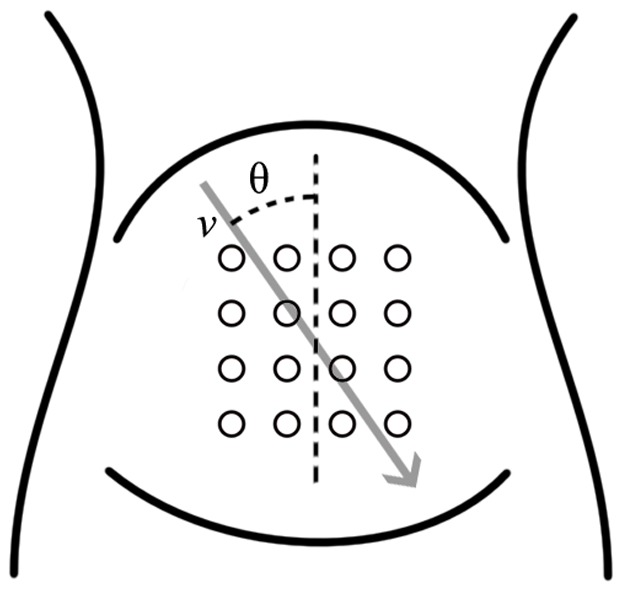
placement and directional definition. Placement of the 16 measuring electrodes. The direction of propagation is defined in relation to the angle θ.

The measurements were performed using a g.GAMMAsys active electrode system (g.tec medical engineering, Schiedlberg, Austria), comprising 16 active electrodes and a biosignal amplifier. The electrodes were fixed in a square four by four grid with an inter-electrode distance of 3.5 cm as shown in figure 1. An active ground electrode, also known as a driven-right-leg electrode, was used to reduce common-mode interference. Double-sided adhesive washers were placed on the electrodes and the electrode grid was placed on the abdominal wall and centered around the umbilicus. A reference electrode was placed on the right hip and the ground electrode was placed halfway between the electrode grid and the reference electrode. All electrodes were filled with a conductive electrode gel of the type g.GAMMAgel (g.tec medical engineering, Schiedlberg, Austria).

**Figure 2:**
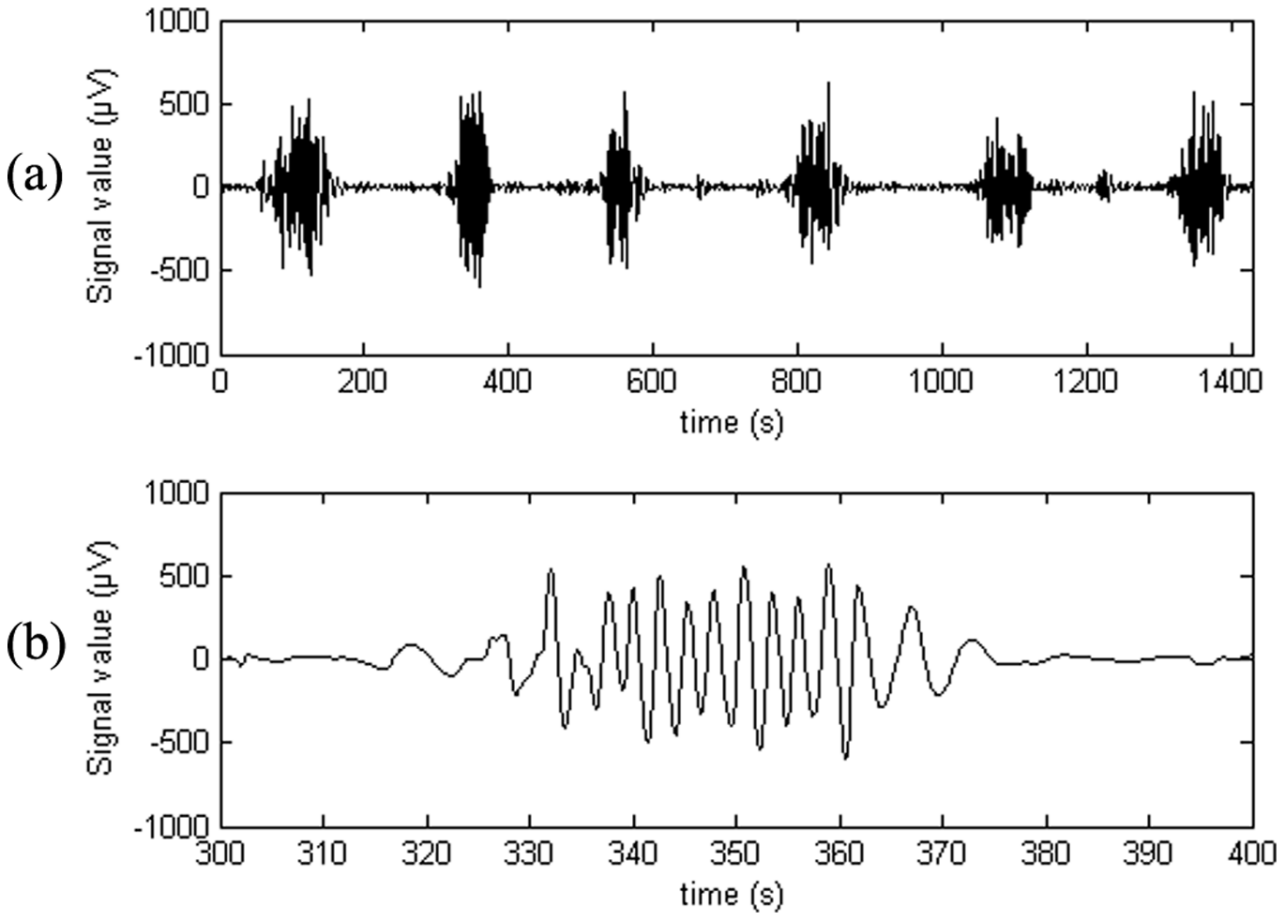
EHG recording. Example of a full EHG recorded by a single electrode (a) and a burst corresponding to a contraction event (b).

The acquired signals were sampled with a sampling frequency of 1200 Hz. The signals used for propagation velocity estimation were down sampled from 1200 Hz to 16 Hz. The EHG signal contains noise from numerous different sources which do not contribute to an understanding of the EHG. The noise sources include drift, fetal and maternal ECG, respiratory movement, EMG interference generated by the contraction of abdominal skeletal muscles, motion artifacts and 50 Hz power line noise. Studies have shown that the frequency content of the maternal ECG can be as low as 1 Hz [15], [24] and showed that the respiratory frequency can be as high as 20 times per minute equivalent to 0.33 Hz. [15] The EMG interference generated by the contraction of abdominal muscles has a dominant frequency component at around 30 Hz. [25] Studies commonly use a filter range from 0.34 Hz to 1 Hz. [13], [15], [26] Other studies use 0.1 Hz to 0.8 Hz. [23], [27] In this study, an upper frequency limit of 1 Hz was chosen. Similarly, a lower frequency limit of 0.1 Hz was used. This introduces the possibility that motion artifacts due to respiration contaminate the signal, but at the same time ensures that the entire EHG signal is recorded. The filter was implemented as a cascade filter of a low-pass and a high-pass eight order Butterworth filter. An example of a filtered and down sampled signal recorded from one electrode is shown in figure 2.

The velocity and direction estimates were computed using a maximum-likelihood approach described by Rabotti *et al.* [23] A brief description of the method will be given here.

The estimates are computed using the signals acquired by a grid composed of *N_r_* rows and *N_c_* columns of electrodes. The EHG signal is assumed to propagate with a fixed velocity *v* and a direction defined by the angle *θ* in respect to the vertical axis of the measuring grid as seen in figure 1. The delay between neighboring rows of electrodes is denoted *τ_r_* and the delay between neighboring columns of electrodes is denoted *τ_c_*. Assuming a fixed velocity and direction of propagation means that all column delays are identical, and all row delays are identical.

The implemented maximum-likelihood algorithm estimates the two delays, *τ_r_* and *τ_c_*, by minimizing the cost function
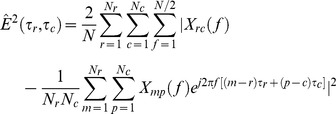
(1)where *X_rc_(f)* is the Discrete Fourier Transform of the signal recorded by the channel in row *r* and column *c*. Similarly, *X_mp_(f)* represents the Fourier transform of the signal recorded by the channel in row *m* and column *p*, and *N* is the length of the DFT. By implementing the cost function in the frequency domain, it can be evaluated for real values of the delay parameters τ_r_ and τ_c_. When the delays have been estimated, the velocity and angle can be computed by the relations



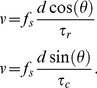
(2)The estimation method was implemented in MATLAB (Mathworks), and the cost function was minimized with a native function using the simplex search method described by Lagarias *et al.* [28] The function was initialized with different delay values to ensure that the detected minimum was also the global minimum.

## Results

**Figure 3:**
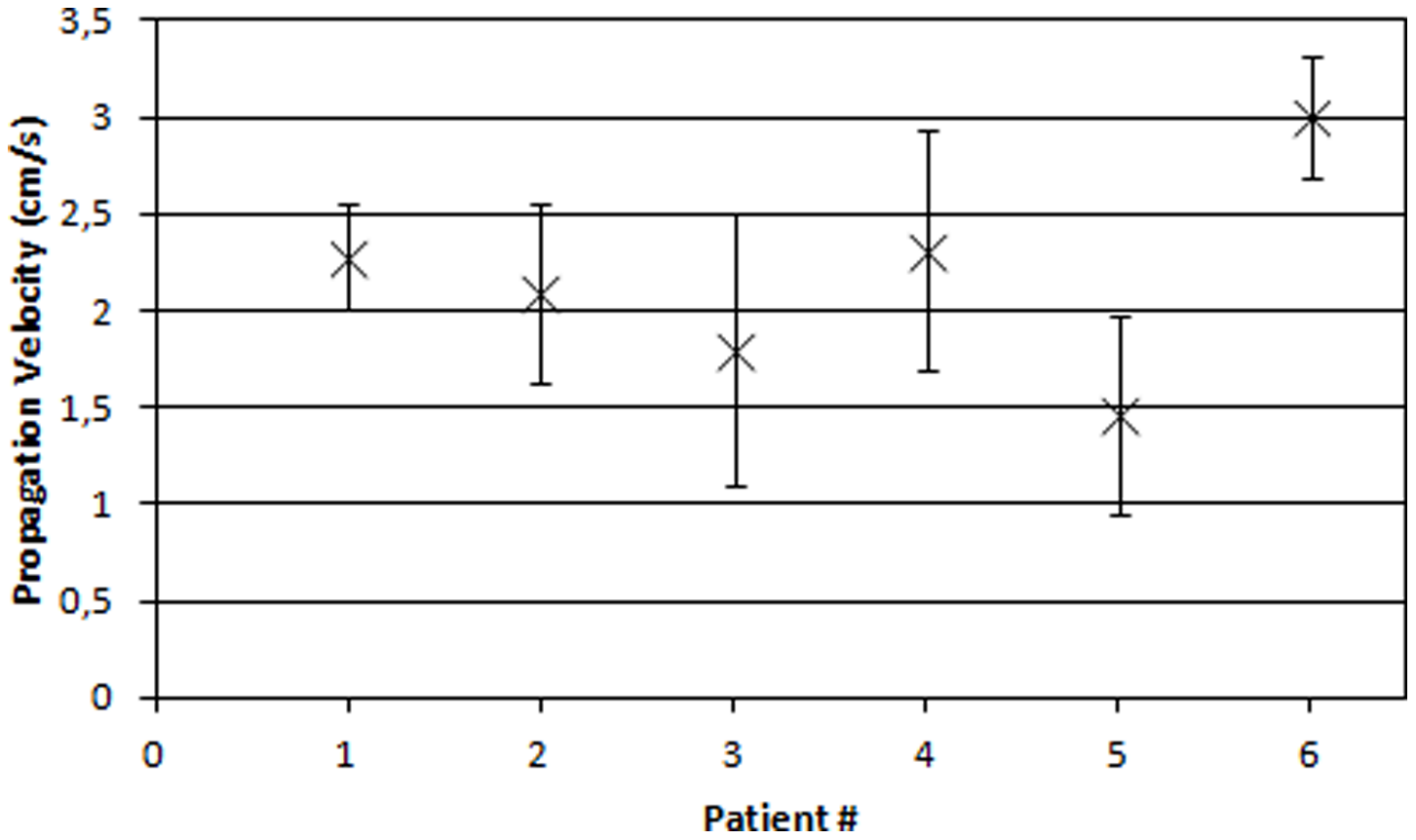
Estimated velocities. Mean and standard deviation of the estimated velocities for all patients.

A total of 35 contractions were extracted from the six measurements from which the direction of propagation and propagation velocities were estimated. Figure 3 shows the average conduction velocities and standard deviations for each patient. The average propagation velocity for all 35 contractions was found equal to 2.18 (±0.68) cm/s.

**Figure 4:**
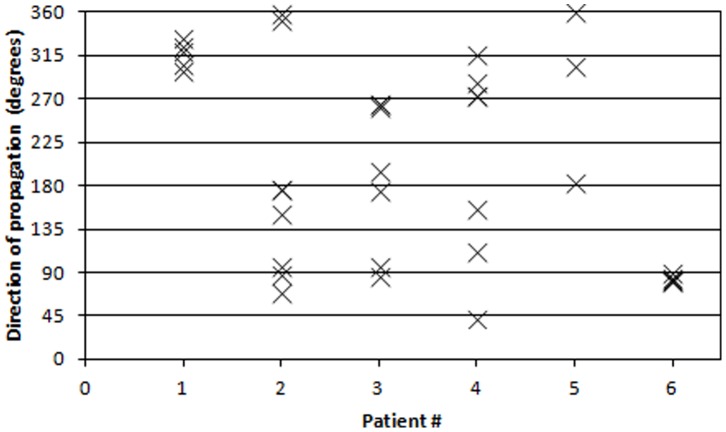
Directional estimates. Distribution of estimated directions of propagation for each patient. Each contraction burst is marked with an X.

**Figure 5:**
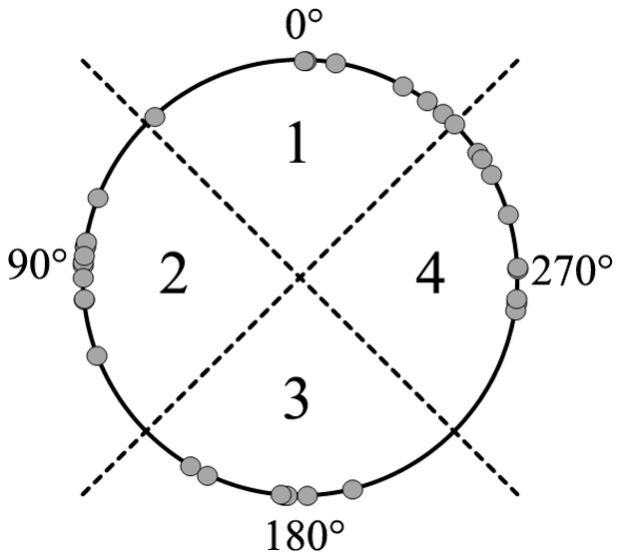
Visualization of the estimated directions of propagation. Direction of propagation is estimated for each contraction and marked with a circle.

**Figure 6:**
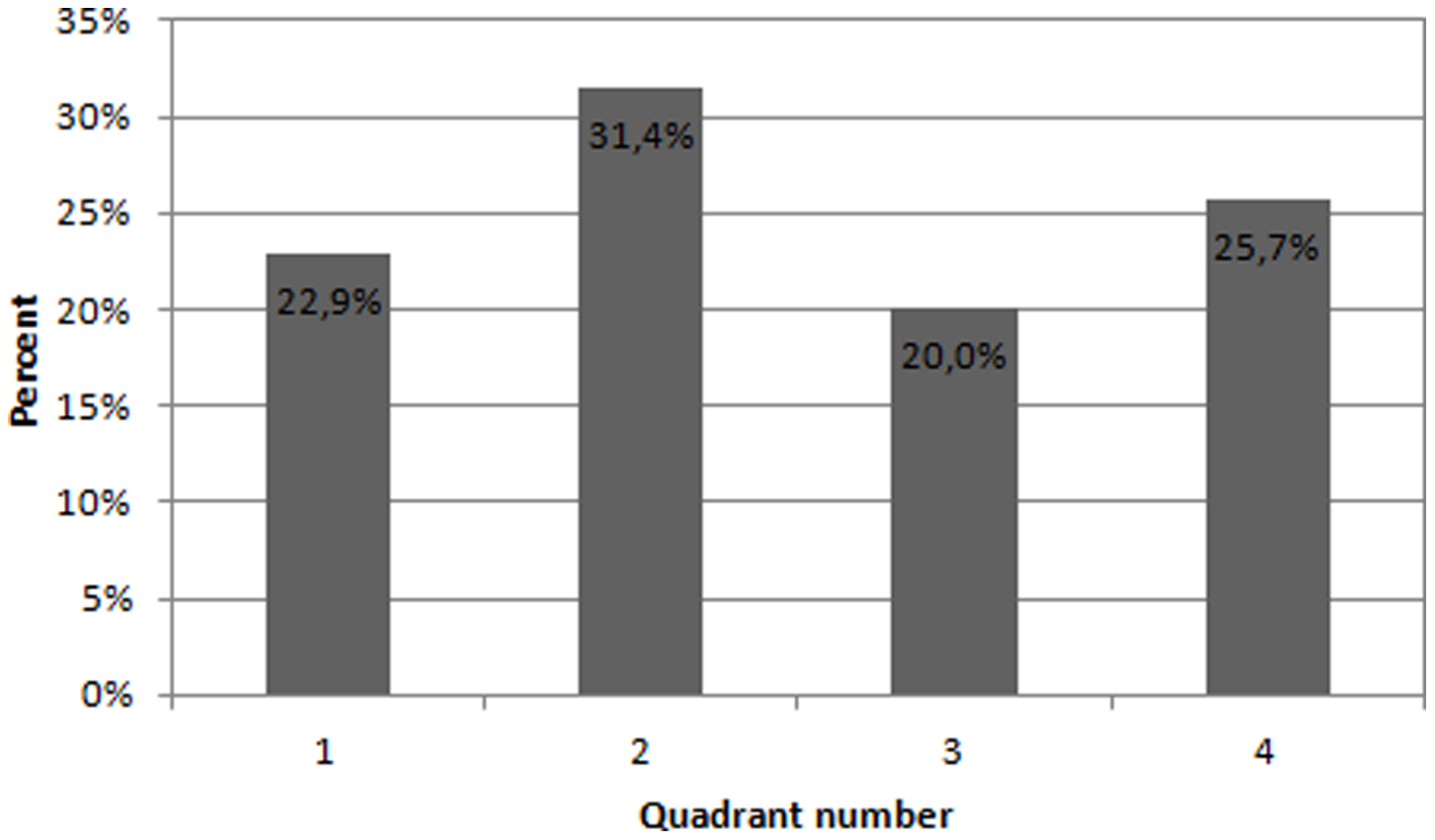
Directional distribution divided into four quadrants. Proportion of contractions with origin in each of the four quadrants.

The estimated direction of propagation can be expressed as an angle in respect to the vertical axis of the measuring grid. This means that an angle of zero degrees corresponds to a contraction originating in the upper part of the uterus with a purely vertical downwards propagation. Similarly, an angle of 180 degrees corresponds to a contraction originating in the lower part of the uterus with a purely vertical upwards propagation. The estimated direction of propagation for each contraction burst is reported in figure 4 for all patients. In an effort to visualize the propagation directions, all 35 contractions are plotted on a circle according to their corresponding angle in figure 5. To see if any preferred direction of propagation exists, the circle is divided into four quadrants corresponding to an upwards, downwards and two sideways directions as also seen in figure 5. The frequency for each directional quadrant is shown in the histogram in figure 6. Dividing the circle into an upper and a lower part, 63% of the contractions originated in the upper part of the uterus and 37% originated in the lower part of the uterus.

## Discussion

In this study we focused on estimating the propagation velocity of entire EHG bursts over a large area of the uterus with a two-dimensional measurement setup. At the same time, the overall direction of propagation was estimated to see if any preferred direction of propagation exists. For many years, EHG measurements have been investigated for the purpose of quantifying uterine activity. Despite this, only a few studies have used two-dimensional measurements to examine the propagation characteristics of EHG signals. [23], [26], [29]

The estimated velocities are comparable to those reported in other EHG studies [22], [23], [30] but with a smaller intra- and inter-patient variation. While other studies report that the estimated velocities are within the physiological expected range, we must stress the fact that, to our knowledge, no previous studies have measured the propagation velocity of electrical signals directly on the human myometrium. These measurements have been performed in several animal studies, [31], [32] but it is uncertain whether these results apply to humans since significant inter-species differences are observed [32]. Nonetheless, the estimated velocities are all within the region previously reported in animal studies and, as such, does not give any reason to question the results.

The results show that no single preferred direction of propagation was found. This corresponds with the results reported from other similar studies, [23], [26] and emphasizes the need for a two-dimensional measuring grid when making estimations of the propagation velocity. When divided into four quadrants, the variations in occurrence of each direction are small as seen in figure 6. The results show that contractions can originate in different parts of the uterus.

Although no single preferred direction of propagation could be identified we did, however, find a seemingly coordinated propagation direction for each contraction in two of the patients. Even though this could potentially be a coincidence it could also be a sign of some sort of coordination of the pacemaking in the uterus. It remains unclear, however, why this coordination is only experienced in two of the six patients (#1 and #6), and it does not seem to be related to the time elapsed between measurement and delivery. This will need to be investigated further.

The mechanisms controlling contraction and relaxation of the uterine muscle cells are today well described. The physiological mechanisms governing generation and propagation of action potentials are, however, not fully understood. For many years the general consent in obstetric textbooks has been that contractions originate in the upper corner of the uterus and propagate downward. [33], [34] This idea is, however, based on a single publication from 1952. [35] Some early EHG studies supported the notion of a preferential downwards propagation of the electrical activity in the uterus during active labor. [36], [37] This supports the notion that there may be general pacemaker regions later in gestation. This theory is disputed by recent EHG studies, showing seemingly random directions of propagation indicating that a contraction can originate in many different areas of the uterus. [23], [26]

Over the recent decades, scientists have identified a type of cells, named interstitial cells of Cajal, which serve as pacemakers for the peristaltic movement of the smooth muscle cells in the gastrointestinal tract. [38] This has led to attempts to find similar cells that act as pacemakers for the human uterus. Myometrial Cajal-like interstitial cells have been identified, but there have been conflicting results as to whether they exhibit spontaneous electrical activity. One study did not find any evidence for a pacemaking role, suggesting that the spontaneous electrical behavior of the myometrium is an inherent property of the smooth muscle cells in the human uterus. [39] In contrast, another study did find spontaneous electrical activity in myometrial Cajal-like interstitial cells. [40] Thus, it remains unclear which cells are responsible for the initiation of contractions, and whether or not any specific pacemaking regions exist. The origin of a contraction can therefore not be predicted. Similarly, the direction of propagation is not known beforehand since the propagation of signals in smooth muscle is not necessarily along the fiber orientation as is the case in skeletal muscle. Additional knowledge about the origin and propagation direction of the electrical activity could provide further insight into the complex physiological mechanisms that control the generation and propagation of contractions.

One of the challenges within this field is the fact that many of the underlying physiological processes are not fully understood. It is not known why and how a contraction is initiated, and there is still no clear description of how a contraction spreads across the uterus. We believe that the key to making accurate contraction assessments with the use of EHG is a more detailed knowledge of the underlying physiological mechanisms governing uterine signal propagation. If these were fully described, a more certain interpretation of the EHG signals would be possible.

In future research studies it should be investigated if a significant difference in conduction velocity can be identified between labor contractions and Braxton Hicks contractions optimally enabling a clinically usable method for predicting preterm labor. It should also be examined if the coordination of the contraction direction from contraction to contraction can be used as an indicator for preterm delivery.
